# Supplementary material for: Long-Term Results of Kidney Transplantation in Patients Aged 60 Years and Older
Source: J Clin Med. 2024 Dec 27;14(1):78. doi: 10.3390/jcm14010078 (PMC11722099; doi:10.3390/jcm14010078)
Supplement: Supplementary file 1 [file jcm-14-00078-s001.zip › jcm-3356509-supplementary.pdf]

Suplementary Table S1. Factors influencing recipient survival in a 10-year follow-up period after kidney transplantation - univariable Cox regression analysis. ≥60 group - recipients aged 60 years and older (study group), <60 group - recipients younger than 60 years (control group)

	<60 group					≥60 group					Stratified				
**Characteristic**	**N**	**Event N**	**HR[1]**	**95% CI[1]**	**p-value**	**N**	**Event N**	**HR[1]**	**95% CI[1]**	**p-value**	**N**	**Event N**	**HR[1]**	**95% CI[1]**	**p-value**
Donor (female)	213	38	0.77	0.39, 1.53	0.463	213	65	1.10	0.67, 1.81	0.708	426	103	0.97	0.65, 1.45	0.882
CVD as reason of donor death	213	38	0.73	0.38, 1.42	0.359	213	65	1.10	0.67, 1.80	0.710	426	103	0.95	0.64, 1.40	0.788
Donor arterial hypertension	213	38	1.21	0.57, 2.56	0.612	213	65	1.37	0.77, 2.45	0.287	426	103	1.31	0.83, 2.07	0.251
Extended criteria donor	213	38	0.89	0.41, 1.95	0.774	213	65	1.80	1.05, 3.10	**0.034**	426	103	1.39	0.89, 2.17	0.147
Multiorgan donor	213	38	0.74	0.39, 1.41	0.368	213	65	0.99	0.59, 1.63	0.954	426	103	0.89	0.60, 1.32	0.551
Induction therapy (yes)	213	38				213	65				426	103			
No			—	—				—	—				—	—	
Induction therapy Simulect			1.16	0.44, 3.06	0.769			1.20	0.65, 2.25	0.558			1.17	0.69, 1.97	0.562
Induction therapy ATG			1.07	0.43, 2.64	0.889			2.02	1.02, 4.00	**0.045**			1.56	0.91, 2.69	0.109
Recipient gender (female)	213	38	0.51	0.25, 1.05	0.068	213	65	0.73	0.43, 1.22	0.226	426	103	0.64	0.42, 0.97	**0.038**
Patients with PRA>25%	213	38	1.16	0.45, 2.99	0.751	213	65	1.29	0.63, 2.62	0.489	426	103	1.24	0.70, 2.19	0.459
Pre-transplant diabetes	213	38	1.91	0.84, 4.34	0.122	213	65	1.48	0.84, 2.60	0.177	426	103	1.60	1.00, 2.55	**0.050**
Any diabetes mellitus	213	38	1.38	0.70, 2.70	0.347	213	65	0.96	0.59, 1.57	0.881	426	103	1.09	0.73, 1.62	0.681
Post transplant diabetes	213	38	0.93	0.39, 2.22	0.864	213	65	0.65	0.34, 1.21	0.174	426	103	0.72	0.43, 1.21	0.217
Recipient CVD	213	38	2.03	0.85, 4.87	0.111	213	65	1.70	1.04, 2.79	0.035	426	103	1.77	1.15, 2.73	0.010
Recipient arterial hypertension	213	38	0.73	0.33, 1.60	0.430	213	65	0.64	0.35, 1.16	0.142	426	103	0.67	0.42, 1.08	0.101
CMV infection	213	38	0.60	0.18, 1.98	0.403	213	65	1.03	0.44, 2.39	0.943	426	103	0.84	0.42, 1.67	0.617
Any acute rejection	213	38	0.61	0.22, 1.71	0.345	213	65	1.52	0.77, 2.99	0.224	426	103	1.08	0.61, 1.90	0.793
Cancer	212	38	1.13	0.44, 2.90	0.803	213	65	1.15	0.63, 2.12	0.646	425	103	1.15	0.69, 1.91	0.602
Surgical complication	213	38	1.98	0.96, 4.07	0.064	213	65	1.0	0.51, 1.95	0.988	426	103	1.32	0.81, 2.16	0.260
Re-transplantation	213	38	1.51	0.71, 3.20	0.280	213	65	3.49	1.57, 7.75	**0.002**	426	103	2.12	1.21, 3.73	**0.009**
Cold ischemia time (h)	213	38	1.00	0.95, 1.05	0.921	213	65	1.05	1.01, 1.08	**0.012**	426	103	1.03	1.00, 1.06	**0.044**
HLA type I mismatch	213	38	1.26	0.92, 1.74	0.151	212	65	1.07	0.85, 1.35	0.556	425	103	1.13	0.94, 1.37	0.184
HLA type II mismatch	213	38	1.11	0.68, 1.80	0.681	213	65	1.30	0.87, 1.94	0.208	426	103	1.22	0.89, 1.66	0.219
Donor age (years)	213	38	0.99	0.96, 1.01	0.293	213	65	1.01	0.99, 1.04	0.233	426	103	1.00	0.99, 1.02	0.779
Recipient BMI (kg/m^2)	210	38	1.05	0.96, 1.15	0.293	205	62	1.03	0.96, 1.10	0.433	415	100	1.04	0.98, 1.10	0.207
Dialysis duration (months)	212	38	1.01	1.00, 1.02	**0.005**	213	65	1.02	1.01, 1.03	**<0.001**	425	103	1.02	1.01, 1.02	**<0.001**
Recipient age (years)	213	38	1.03	1.00, 1.06	0.065	213	65	1.05	0.98, 1.13	0.172	–	–	–	–	–

[1]HR = Hazard Ratio, CI = Confidence Interval

Suplementary Table S2. Factors influencing kidney graft survival in a 10-year follow-up period after kidney transplantation - univariable Cox regression analysis. ≥60 group - recipients aged 60 years and older (study group), <60 group - recipients younger than 60 years (control group)

	**<60 group**					**≥60 group**					**Stratified**				
**Characteristic**	**N**	**Event N**	**HR[1]**	**95% CI[1]**	**p-value**	**N**	**Event N**	**HR[1]**	**95% CI[1]**	**p-value**	**N**	**Event N**	**HR[1]**	**95% CI[1]**	**p-value**
Donor (female)	213	73	0.94	0.58, 1.51	0.785	213	84	1.19	0.77, 1.84	0.438	426	157	1.06	0.77, 1.47	0.704
CVD as reason of donor death	213	73	0.78	0.49, 1.25	0.299	213	84	1.22	0.79, 1.88	0.359	426	157	0.99	0.72, 1.36	0.961
Donor arterial hypertension	213	73	1.01	0.57, 1.78	0.983	213	84	1.52	0.92, 2.52	0.104	426	157	1.25	0.86, 1.83	0.242
Extended criteria donor	213	73	1.24	0.73, 2.12	0.424	213	84	2.59	1.64, 4.09	**<0.001**	426	157	1.85	1.31, 2.61	**<0.001**
Multiorgan donor	213	73	0.58	0.37, 0.92	**0.021**	213	84	0.86	0.55, 1.33	0.496	426	157	0.71	0.52, 0.98	**0.038**
Induction therapy (yes)	213	73				213	84				426	157			
No			—	—				—	—				—	—	
Induction therapy Simulect			1.01	0.49, 2.08	0.973			0.99	0.56, 1.77	0.985			0.98	0.63, 1.54	0.937
Induction therapy ATG			1.14	0.61, 2.13	0.685			1.94	1.08, 3.48	**0.026**			1.49	0.97, 2.28	0.066
Recipient gender (female)	213	73	0.58	0.35, 0.96	**0.034**	213	84	0.77	0.49, 1.21	0.255	426	157	0.67	0.48, 0.94	**0.022**
Patients with PRA>25%	213	73	1.47	0.79, 2.73	0.225	213	84	1.29	0.70, 2.40	0.415	426	157	1.38	0.89, 2.13	0.153
Pre-transplant diabetes	213	73	1.41	0.74, 2.67	0.298	213	84	1.47	0.90, 2.42	0.125	426	157	1.45	0.98, 2.14	0.064
Any diabetes mellitus	213	73	0.93	0.55, 1.56	0.775	213	84	0.89	0.58, 1.38	0.608	426	157	0.91	0.65, 1.27	0.564
Post transplant diabetes	213	73	0.65	0.32, 1.31	0.230	213	84	0.57	0.32, 1.01	0.053	426	157	0.60	0.38, 0.93	**0.024**
Recipient CVD	213	73	1.54	0.77, 3.10	0.225	213	84	1.42	0.91, 2.21	0.120	426	157	1.45	1.00, 2.11	0.051
Recipient arterial hypertension	213	73	0.73	0.41, 1.29	0.279	213	84	0.80	0.46, 1.38	0.419	426	157	0.76	0.51, 1.14	0.183
CMV infection	213	73	0.82	0.38, 1.81	0.630	213	84	0.96	0.44, 2.09	0.924	426	157	0.89	0.51, 1.55	0.678
Any acute rejection	213	73	2.67	1.61, 4.44	**<0.001**	213	84	1.26	0.67, 2.39	0.472	426	157	1.92	1.30, 2.83	**<0.001**
Cancer	212	72	0.60	0.26, 1.40	0.240	213	84	1.12	0.66, 1.91	0.674	425	156	0.91	0.58, 1.42	0.680
Surgical complication	213	73	2.21	1.31, 3.72	**0.003**	213	84	1.71	1.00, 2.91	**0.050**	426	157	1.94	1.33, 2.81	**<0.001**
Re-transplantation	213	73	1.81	1.08, 3.04	**0.024**	213	84	3.09	1.53, 6.25	**0.002**	426	157	2.14	1.40, 3.27	**<0.001**
Cold ischemia time (h)	213	73	1.02	0.98, 1.06	0.313	213	84	1.04	1.01, 1.07	**0.012**	426	157	1.03	1.01, 1.06	**0.010**
HLA type I mismatch	213	73	1.23	0.98, 1.55	0.068	212	84	1.11	0.91, 1.37	0.307	425	157	1.17	1.00, 1.36	**0.047**
HLA type II mismatch	213	73	1.31	0.92, 1.87	0.140	213	84	1.47	1.04, 2.09	**0.031**	426	157	1.39	1.08, 1.78	**0.010**
Donor age (years)	213	73	1.02	1.00, 1.04	0.103	213	84	1.02	1.00, 1.05	**0.023**	426	157	1.02	1.01, 1.04	**0.005**
Recipient BMI (kg/m^2)	210	72	1.03	0.96, 1.10	0.414	205	80	1.03	0.97, 1.10	0.348	–	–	–	–	**–**
Dialysis duration (months)	212	73	1.01	1.00, 1.02	**0.025**	213	84	1.02	1.01, 1.03	**<0.001**	425	157	1.01	1.01, 1.02	**<0.001**
Recipient age (years)	213	73	1.01	0.99, 1.03	0.551	213	84	1.03	0.97, 1.10	0.285	426	157	1.01	0.99, 1.03	0.373

[1]HR = Hazard Ratio, CI = Confidence Interval

Suplementary Table S3. Factors influencing death censored kidney graft survival in a 10-year follow-up period after kidney transplantation - univariable Cox regression analysis. ≥60 group - recipients aged 60 years and older (study group), <60 group - recipients younger than 60 years (control group)

	<60 group					≥60 group					Stratified				
**Characteristic**	**N**	**Event N**	**HR**[1]	**95% CI**[1]	**p-value**	**N**	**Event N**	**HR**[1]	**95% CI**[1]	**p-value**	**N**	**Event N**	**HR**[1]	**95% CI**[1]	**p-value**
Donor (female)	213	46	0.98	0.54, 1.78	0.943	213	27	1.40	0.65, 2.99	0.388	426	73	1.12	0.70, 1.79	0.639
CVD as reason of donor death	213	46	0.80	0.44, 1.45	0.459	213	27	1.78	0.83, 3.80	0.139	426	73	1.08	0.68, 1.71	0.754
Donor arterial hypertension	213	46	1.08	0.54, 2.18	0.830	213	27	1.61	0.68, 3.82	0.278	426	73	1.25	0.73, 2.16	0.414
Extended criteria donor	213	46	1.67	0.89, 3.12	0.112	213	27	4.52	2.12, 9.65	**<0.001**	426	73	2.44	1.52, 3.93	**<0.001**
Multiorgan donor	213	46	0.50	0.28, 0.90	**0.020**	213	27	0.56	0.26, 1.19	0.129	426	73	0.52	0.33, 0.83	**0.006**
Induction therapy (yes)	213	46				213	27				426	73			
Induction therapy Simulect			0.85	0.33, 2.20	0.735			0.31	0.07, 1.33	0.115			0.56	0.25, 1.26	0.161
Induction therapy ATG			0.94	0.41, 2.16	0.875			1.53	0.58, 4.01	0.386			1.14	0.61, 2.13	0.685
Recipient gender (female)	213	46	0.77	0.42, 1.41	0.392	213	27	1.05	0.49, 2.26	0.905	426	73	0.86	0.54, 1.39	0.541
Patients with PRA>25%	213	46	1.34	0.60, 3.00	0.480	213	27	2.20	0.88, 5.51	0.094	426	73	1.63	0.89, 2.98	0.112
Pre-transplant diabetes	213	46	1.19	0.51, 2.81	0.689	213	27	0.98	0.37, 2.59	0.964	426	73	1.09	0.57, 2.08	0.795
Any diabetes mellitus	213	46	0.68	0.34, 1.38	0.290	213	27	0.75	0.34, 1.65	0.479	426	73	0.71	0.42, 1.20	0.206
Post transplant diabetes	213	46	0.44	0.16, 1.24	0.120	213	27	0.68	0.26, 1.81	0.439	426	73	0.55	0.27, 1.10	0.091
Recipient CVD	213	46	1.04	0.37, 2.91	0.935	213	27	0.80	0.34, 1.88	0.605	426	73	0.89	0.45, 1.73	0.721
Recipient arterial hypertension	213	46	0.66	0.32, 1.34	0.248	213	27	2.68	0.63, 11.4	0.182	426	73	0.99	0.53, 1.85	0.965
CMV infection	213	46	1.18	0.50, 2.80	0.709	213	27	0.86	0.20, 3.66	0.843	426	73	1.08	0.51, 2.26	0.843
Any acute rejection	213	46	4.35	2.39, 7.90	**<0.001**	213	27	0.63	0.15, 2.68	0.534	426	73	2.71	1.62, 4.52	**<0.001**
Cancer	212	45	0.15	0.02, 1.10	0.062	213	27	1.07	0.40, 2.82	0.898	425	72	0.52	0.23, 1.21	0.131
Surgical complication	213	46	2.21	1.14, 4.28	**0.018**	213	27	3.28	1.47, 7.31	**0.004**	426	73	2.57	1.55, 4.28	**<0.001**
Re-transplantation	213	46	2.12	1.13, 3.98	**0.020**	213	27	1.83	0.43, 7.88	0.417	426	73	2.07	1.16, 3.68	**0.013**
Cold ischemia time (h)	213	46	1.03	0.98, 1.08	0.229	213	27	1.02	0.97, 1.08	0.429	426	73	1.03	0.99, 1.06	0.152
HLA type I mismatch	213	46	1.27	0.96, 1.69	0.098	212	27	1.39	0.95, 2.03	0.087	425	73	1.31	1.05, 1.65	**0.018**
HLA type II mismatch	213	46	1.56	1.01, 2.43	**0.047**	213	27	1.88	1.02, 3.45	**0.043**	426	73	1.66	1.16, 2.38	**0.005**
Donor age (years)	213	46	1.05	1.01, 1.08	**0.005**	213	27	1.04	1.00, 1.08	**0.045**	426	73	1.04	1.02, 1.07	**<0.001**
Recipient BMI (kg/m^2)	210	45	1.02	0.94, 1.11	0.664	205	25	1.04	0.93, 1.16	0.542	415	70	1.02	0.96, 1.10	0.474
Dialysis duration (months)	212	46	1.01	1.00, 1.02	0.242	213	27	1.02	1.01, 1.03	**0.004**	425	73	1.01	1.00, 1.02	**0.011**
Recipient age (years)	213	46	0.99	0.96, 1.01	0.398	213	27	0.93	0.82, 1.05	0.241	–	–	–	–	**–**

[1]HR = Hazard Ratio, CI = Confidence Interval
